# Exploring the decision to disclose the use of natural products among outpatients: a mixed-method study

**DOI:** 10.1186/1472-6882-13-319

**Published:** 2013-11-18

**Authors:** Hsiao-Yun Chang, Huai-Lu Chang, Betty Siren

**Affiliations:** 1 School of Nursing, Fooyin University, Kaohsiung, Taiwan; 2 Chief Executive Officer, H & L Medical Corporation, Kaohsiung, Taiwan; 3 Department of Foreign Languages, Fooyin University, Kaohsiung, Taiwan

**Keywords:** Natural products, Disclosure, Communication, Decision, Mixed methods research

## Abstract

**Background:**

There is little understanding of the reasons for the limited communication between patients and conventional healthcare professionals regarding patients’ use of complementary and alternative medicine (CAM). The purpose of this study is to explore the predictors of outpatients’ decision to disclose their use of natural products to conventional healthcare professionals.

**Methods:**

A mixed method design was used. Quantitative data were obtained through a survey and qualitative data were obtained from semi-structured interviews. A total of 257 outpatients who fulfilled the criteria of having used natural products prior to the interview were recruited for this study. Subsequently, 39 patients of those who completed the survey were further selected to take part in an in-depth qualitative interview.

**Results:**

Predictors of the decision to disclose the use of natural products to conventional healthcare professionals included age, frequency of clinic visits, knowledge of the natural products and the attitude towards the benefits of CAM use. The themes that emerged from the qualitative data included safeness of the natural products, consulting alternative sources of information, apprehension regarding the development of negative relationships with healthcare professionals and reactions from the healthcare professionals.

**Conclusions:**

Understanding the factors and reasons affecting patients’ decision as to whether to disclose their use of natural products provides an opportunity for conventional healthcare professionals to communicate better with patients. It is important to encourage patients to disclose their use of natural products in order to provide responsible health care as well as increasing patient safety regarding medication usage.

## Background

Complementary and alternative medicine (CAM) refers to a set of healthcare beliefs, approaches, and products that are infrequently taught to conventional healthcare students, and are not generally available at hospitals [1,2]. When compared to Western countries such as the USA (38.3%) [3] and Australia (52.2%) [4], rates of CAM use are exceptionally high among individuals from countries with long histories such as Singapore (76%) [5], Japan (76%) [6], Turkey (70%) [7] and Taiwan (75.5%) [8]. Most users of CAM treatment are often resistant towards abandoning CAM in favour of conventional treatments, but rather preferring a range of available options that complement conventional medicine [9]. As such, many CAM users do not divulge information about their CAM use to conventional healthcare professionals. Recent studies showed the overall rate of non-disclosure of CAM use remains high, between 48.2% and 73.4% [9-11]. In addition, the decision on whether to disclose CAM use to conventional healthcare professionals also varies among patient populations. In the US, studies found that only 39.8% of CAM users disclosed their use with physicians in 1990 and, in 1997, an even lower 38.5% disclosed this information [12,13]. The highest rate of non-disclosure in CAM use was found in patients with end-stage renal disease (ESRD) receiving haemodialysis in Taiwan, where 98% of the CAM users did not disclose their CAM use to their primary care physicians [14]. Although previous studies have extensively explored the rates of disclosure or non-disclosure of CAM use to conventional healthcare professionals, there is little understanding of the reasons for this limited communication and the factors influencing this gap in communication.

The use of natural products within CAM has been shown to be a popular choice of therapy among patients [15,16] where many people hold the misconception that these products are natural, and hence, safe to use without adverse effects. In reality, the addition of herbal medicines to conventional treatment regimens holds the potential of herb-drug interactions [17]. Natural products are classified as biologically based therapies, including herbs, botanicals, vitamins and minerals, and dietary supplements. With high use but limited disclosure of CAM use, patients may impose on themselves risks such as making uninformed decisions regarding other treatments involving drug interactions, and misdiagnosis and mismanagement of their disease. Serious complications may develop as a result, affecting the course of their disease. For example, Leung et al. [18] found that 34.1% of natural products users who consumed herbs, botanicals, vitamins and supplements orally while on warfarin therapy were at risk of excessive bleeding. Some natural products may also cause interactions with anticancer drugs by inducting drug-metabolizing enzymes and ATP-binding cassette drug transporters, which lower plasma levels of the anticancer drugs and may lead to cancer treatment failure. Some examples of natural products that could cause adverse effects in anticancer therapy are kava-kava, vitamin E, quercetin, ginseng, garlic, beta-carotene and Echinacea [19]. This withholding of information between patients and conventional healthcare professionals increases the possibility of serious herb-drug interactions occurring which may have a negative impact on the patients’ health. However, the decision of patients to withhold disclosure about their use of natural products remains a complex issue to be explored. There is an urgent need to understand the profile characteristics of patients who are likely to disclose the use of natural products versus those who are unlikely to do so, as well as the reasons for which patients hesitate to disclose this information. Therefore, the purpose of this study is to shed light on 1) the reasons some patients choose to disclose their use of natural products to conventional healthcare professionals while others choose not to, and 2) the factors associated with these decisions.

## Methods

### Design

This study employed a mixed method design integrating both quantitative and qualitative methods. This approach of qualitative-quantitative methodological triangulation reconciles the two methods which embody incompatible assumptions [20]. In this study, a sequential mixed-model design was used, incorporating quantitative data obtained from surveys with qualitative data from semi-structured interviews to better explore the phenomena that could not be quantified. This model design use qualitative data to explain in more detail of quantitative findings and help to better understand how the personal experiences of disclosure about the use of natural products to conventional healthcare professionals.

### Sample

A sample of patients who attended medical and surgical clinics at the Zuoying Armed Forces General Hospital in Taiwan between August 2007 and August 2008 were invited to participate. To qualify for inclusion, patients had to meet the following criteria: 1) over the age of 18, 2) using at least one natural products in the past 12 months and 3) sufficient ability to understand and respond to all questions on the survey instrument. A total of 521 patients were screened for CAM use and 358 patients (68.73%) reported using CAM. However, only 257 patients who reached the inclusion criteria of having used natural products prior to the study were recruited. The patients from the qualitative phase of the study were selected from the overall pool of patients in the quantitative phase. A purposive sampling strategy was used for this phase in order to select those who were able to discuss their decision about the disclosure of natural products use, came from diverse demographic backgrounds and were information rich [21]. In the qualitative phase, we recruited a total of 39 patients, and conducted interviews until there was data saturation—in other words, when further sampling yielded no new information [22].

### Instrument & interview guide

The survey was divided into three sections relating to the sample characteristics, information about their use of natural products and attitudes towards the use of CAM. Sample characteristics collected included demographic and clinical information about the patients’ age, gender, education level, occupation, marital status, duration of disease, frequency of clinic visits and level of medication compliance. Information about the use of natural products included how to mix use of natural products and conventional treatment, knowledge of natural products, and whether patients disclosed their use of natural products to conventional healthcare professionals.

Patients’ attitudes towards CAM use was assessed using the scales modified from the works of Hsu et al. [23] and Chang et al. [24] in which the scale focused on the perceived benefits of and perceived barriers to the use of CAM for depression and diabetes, respectively. In our scale, the wordings related to depression or diabetes were deleted so that items suited all outpatients. A 20-item scale enquiring on a 4-point Likert scale from ‘strongly disagree’ to ‘strongly agree’ was used to assess the attitudes of patients towards CAM use, where a higher score indicates a positive attitude toward CAM and lower score hold a negative attitude towards CAM. Cronbach’s alpha, a measure of internal consistency reliability, was 0.8–0.92 in both studies. Cronbach’s alpha in the present study showed acceptable reliability for both the benefits (α = .87) and the barriers (α = .91) of CAM use. The content validity of the instrument was established by a panel of experts. Three academic healthcare professionals and two nurses who were either experts in CAM research, conventional health care or research methods evaluated the instrument and minor adjustments were made.

A semi-structured interview guide was developed for the qualitative research to answer the research question ‘What is your experience with regard to discussing your use of natural products with conventional healthcare professionals?’ To facilitate the interview, patients were probed to further elaborate on describing their decisions to disclose the use of natural products or to clarify areas of confusion. The interview guide was reviewed by a panel of experts. Qualitative rigour was ensured in the follow ways: (1) verbatim recording, transcribing of recordings and writing of notes during the process of interview were carried out and (2) any paraphrasing of interview content done on the part of the researchers was verified when the patients came down to the clinics for during their next appointment. This happened for two of the patients. Feedback from these patients confirmed that most of the findings presented their point of view and only clarification on minor misunderstandings of patients’ words was done because of the use of dialect during the interview.

### Ethical consideration

The study was approved by the hospital ethics committee for human subjects and followed the Declaration of Helsinki. Patients were given an information sheet explaining the research and the ethical considerations, and then signed informed consent forms. All patients were informed that a refusal or a termination of their participation would not have any adverse consequence on their care within the facility. Patient codes were used, instead of their names, to ensure confidentiality.

### Statistical analyses

The quantitative data were scanned for completeness and responses were coded and entered into the computer using IBM^®^ SPSS^®^ for Windows Version 17.0. Demographic characteristics and clinical data were summarised through descriptive statistical procedures. Comparisons were made between ‘disclosers’ and ‘non-disclosers’ using inferential statistics. Stepwise multiple logistic regression with the Hosmer–Lemeshow goodness-of-fit tests was performed to explore the factors associated with the disclosure of the use of natural products. The significance threshold for all analyses was 0.05.

Qualitative data from the interview were analysed by using the three-step coding method: (1) open coding, (2) axial coding and (3) selective coding [25]. In order to strengthen the reliability of the interpretations and findings, the raw data were analysed in Chinese by two separate researchers to ensure findings that are authentic, original, and reliable. After completing the coding, the researchers discussed their individual findings to detect potential biases or inappropriate subjectivity and all disagreements were discussed until a consensus was achieved. Then, the researchers chose categories and subcategories which best described the experience of each patient regarding the decision of whether to disclose the use of natural products to conventional healthcare professionals.

## Results

### Characteristics of sample

A total of 257 patients were recruited in the quantitative phase, including 190 people who chose not to disclose the use of natural products and 67 people who chose to disclose. The rate of non-disclosure in this study is 74%. Sample demographic and clinical data are presented in Table 1, delineated along patients who chose to disclose the use of natural products or not. The mean age of the patients was 54 years (*SD* = 15 years; range: 20–87), and the mean duration of their disease was 6.5 years (*SD* = 6.8 years; range: 1 month to 35 years). The majority of patients had completed at least high school (68.5%), were married (60.3%), were retired (40.1%), visited the clinic monthly (63.0%), and higher medication compliance (75.5%). Among the 257 patients who used natural products, only 26% (*n* = 67) disclosed their use to their healthcare professionals. In the qualitative phase, 39 of these patients were interviewed (14 men and 25 women; mean age = 60 years; range: 38–87). Their education levels ranged from primary school education to a master’s degree.

**Table 1:** Comparisons between non-disclosers and disclosers

**Variables**	**Non- Disclosers n = 190**		**Disclosers n = 67**		***t*-test**	***p *** **value**
	**Mean**	**SD**^**a**^	**Mean**	**SD**		
**Age**	55.5	15.3	50.9	15.0	2.15	0.032
**Duration of disease**	1.70	1.45	1.36	1.48	1.67	0.091
				**Mann–Whitney *U *** **test**		
**Benefits of CAM**^**b **^**use**	25.5	5.8	26.7	5.3	-2.10	0.036
**Barriers of CAM use**	24.3	6.1	25.2	5.8	-1.05	0.295
	**n**	**%**	**n**	**%**	**Chi-square**	
**Gender**					0.09	0.771
male	84	44.2	31	46.3		
female	106	55.8	36	53.7		
**Education**					1.59	0.208
<high school	64	33.6	17	25.4		
>high school	126	66.3	50	74.7		
**Occupation**					0.33	0.847
working	70	36.8	27	40.3		
not working	42	22.1	15	22.4		
retired	78	41.1	25	37.3		
**Marriage**					3.58	0.310
single	47	24.7	18	26.9		
married or partner	111	58.4	44	65.7		
divorce	5	2.6	1	1.5		
wifeless or widow	27	14.2	4	6.0		
**Clinic visited**					6.81	0.033
fortnightly	14	7.4	1	1.5		
monthly	124	65.3	38	56.7		
seasonally	52	27.3	28	41.8		
**Medication compliance**					1.89	0.596
not taken	26	13.7	13	19.4		
sometime taken	7	3.7	2	3.0		
most time taken	10	5.3	5	7.5		
always taken	147	77.4	47	70.1		
**How to mixed with conventional medicines**					8.74	0.068
no change	51	26.8	26	38.8		
separately taken	106	55.8	38	56.7		
reduce medicine dose	8	4.2	0	.0		
stop medicine	12	6.3	1	1.5		
others	13	6.8	2	3.0		
**Knowledge of natural products**					11.50	0.001
unknown	132	69.5	31	46.2		
known	58	30.5	36	53.7		

a = Standard deviation; b = Complementary and alternative medicine.

### Differences in attitudes toward CAM use between disclosers and non-disclosers

The results indicated that patients who disclose the use of natural products had higher total scores on the perceived benefits of CAM use than people who chose not to disclose (Z = -2.10, *p* = .036). Within the scale of the perceived benefits of CAM use, the disclosers strongly believed that CAM can relieve symptoms (Z = -2.86, *p* = .004) and improve energy (Z = -2.80, *p* = .005) and believed that their CAM use decreases the need for conventional medications (Z = -2.49, *p* = .013). However, no significant difference was noted on the attitudes towards the barriers of CAM use between disclosers and non-disclosers (Z = -1.05, *p >* .295).

### Predictors of disclosure in using natural products

According to the results presented in Tables 1 and 2, significant differences between non-disclosers and disclosers were found in relation to age (*t* = 2.15, *p* = .032), frequency of clinic visits (χ^*2*^ = 6.81, *p* = .033), the proportions of patients who know the ingredients of the natural products (χ^*2*^ = 11.50, *p* = .001), and the perception of benefits (attitudes) of CAM use (*Z* = -2.1, *p* = .036). All the variables and scale items were entered into the multiple logistic regression analysis. The findings showed that the knowledge of natural products (OR = 2.63, CI = 1.43–4.85) was the strongest factor associated with disclosure, followed by the belief that CAM use can improve energy (OR = 1.88, CI = 1.10-3.22), believing that CAM use can strengthen the conventional medicine (OR = 1.86, CI = 1.08-3.19) and the frequency of clinic visits (OR = 1.57, CI = 1.15-2.67). Only one variable is reverse from others which is that CAM user believe better to use one alone (OR = 0.35, CI = 0.19-0.64).

**Table 2:** Predictors of disclosing natural products use

**Factors**	**B**	**S.E.**	**Exp(B)**	**95% C.I.**		***p***
				**Lower**	**Upper**	
The knowledge of CAM components	1.00	.31	2.71	1.47	5.01	.001
The improvement of energy	.68	.26	1.97	1.18	3.30	.010
The strength of my conventional medicine	.59	.30	1.81	1.00	3.27	.050
The frequency of clinic visits	.37	.15	1.44	1.07	1.95	.017
Better to use one alone	-1.06	.31	.35	.19	.64	.001

1. Hosmer and Lemeshow Test (*X*^*2*^ = 5.61, p = .69); 2. Cox & Snell R Square = .127 & Nagelkerke R Square = .186.

### Qualitative data – The decision regarding the disclosure of using natural products

The decision on whether or not to disclose the use of natural products to conventional healthcare professionals is a very personal matter. During the semi-structured interviews, patients who did not disclose their natural products use were asked about their reasons and patients who chose to disclose their use of natural products were asked about their feelings regarding the responses and reactions received from their professionals. Results from the interviews indicated that 26 patients who did not disclose the use of natural products felt that disclosure was not necessary for the following reasons: they felt that the use of natural products were safe, they had consulted others about the safety of the natural products and they feared that they might develop a negative relationship with their conventional heath care professionals. On the other hand, 13 patients who disclosed the use of natural products reported that the responses from conventional healthcare professionals were not favourable. Patients’ experiences were that they felt being pushed away from the use of natural products, that the safety of its usage was entirely their own responsibility or that the communication was generally unclear or dismissive. All the categories are listed in Table 3.

**Table 3:** Findings from qualitative data

**Themes**	**Categories**
Feeling safe with the use of natural products	• Adjusting taken both medicines
	• No interaction with conventional medicine
Consulting acquaintances having medical knowledge	• Friends
	• Family & relatives
	• CAM practitioners
Fear of a negative relationship development	• Anticipating a negative attitude from conventional healthcare professional
	• Affecting a trust relationship between conventional healthcare professional and patients
	• Lack of knowledge in the field of natural products.
Experience of responses	• Discouraging them from using it
	• Our own responsibility for the safety of its usage
	• Unclear or dismissive communication

Patients who chose not to disclose felt that natural products were safe to use. Their methods included consuming the natural products at a different time from their conventional medicine, lowering the dosage of the conventional medicine or taking natural products that do not target the treatment of their primary medical condition. These rationalisations helped them feel that there were no safety issues related to the use of the natural products. One patient related, ‘I took them separately…, I think it is safe, so I did not discuss with the conventional healthcare professional’ (P1). Several patients believed that some natural products such as nutritional supplements have no side effects or interaction with conventional medicine. When asked if they were afraid of combining the two remedies, they replied, ‘It is a health product (nutritional supplement) and has no interaction with conventional medicine’ (P12).

Patients who felt that using the natural products were safe did not feel that there is a need to discuss this with their conventional healthcare professionals. However, when people felt uncomfortable or sceptical about the products, they would seek advice from their family members or talk to acquaintances who worked in the medical field; for example, ‘I also have a friend who is a sales representative for pharmacies. Before I tried this product, I asked my friend to check it and she said it was all right’ (P33). The findings show that when patients had questions about natural products, they usually made enquiries about it from their friends whose work had some relation to the medical field, but they seldom raised those concerns with their conventional healthcare professionals.

Patients expected a negative relationship would develop with the disclosure of their natural products usage to conventional healthcare professionals, including being rejected or reprimanded by the conventional healthcare professional, having a breakdown of trust between patient and conventional healthcare professional and the fear of a negative attitude held towards them. In general, a recurring point that emerged is that conventional healthcare professionals hold negative perspectives about their use of natural products. The negative reaction anticipated during medical consultation was a strong factor leading to the avoidance in discussion about their use of natural products. Patient 4 revealed, ‘The biggest reason is that I am afraid of being scolded by a conventional healthcare professional.’ Patient 15 explained, ‘I have never discussed, because some of the conventional healthcare professionals will reject it (natural products) and also scold you said ‘if you are so good at this, you can just treat yourself.’ It depends on the degree of (the) conventional healthcare professional’s acceptance’ (P15). In addition, many patients also complained about the difficulties in discussing natural products with those professionals. They reported that conventional healthcare professionals looked ‘unhappy’ (P30), ‘not happy’ (P2), and ‘not too happy’ (P13).

Patients were also afraid that if they disclose their use of natural products, the level of patient-conventional healthcare professional trust would change. For example:

If you told a conventional healthcare professional about this, he would say you don’t believe him, simply don’t come back to clinic. I am terrified if he said like this. Some of the healthcare professionals are so that (mean)… he (conventional healthcare professional) would say ‘this is inappropriate, you already visit conventional healthcare professional with a good health outcome, why you need to see TCM (traditional Chinese medicine) practitioner at the same time.’ It let you feel you actually don’t believe in conventional medicine (P26).

In the presence of the conventional healthcare professional as the authoritative figure, many patients experienced a fear of disclosing their use of natural products during consultations. However, in order to avoid any adverse reactions occurring with the use of both natural products and conventional medicines, patients were more willing to disclose the issue to their CAM practitioners. This can be observed in patient 37 who reported, ‘if the same situation (the disclosure of conventional medicine) happened with a CAM practitioner, he would be able to incorporate his medicine with the conventional medicine.’ In addition to fear, the patients felt that conventional healthcare professionals do not have adequate knowledge about the natural products to respond to their concerns and this was highlighted by patient 8:

*‘*Because you (conventional healthcare professionals) don’t understand the Chinese herbal medicine comparatively…People have their own speciality, so I asked him [CAM practitioner].

Our findings revealed that patients’ sense of safety regarding the use of the natural products, consultation of others in related fields, and fear of negative reactions from the professionals were major considerations in the non-disclosure of using natural products. Additionally, insufficient knowledge regarding natural products on the part of the conventional healthcare professionals also contributed to this lack of disclosure.

For 13 patients who disclosed their use of natural products, it was revealed that they experienced the feeling of being pushed away from the use of natural products, were told that adverse effects of consumption fall within their own responsibility, they felt they were being discouraged from continuing its use and that communication regarding their use of natural products was not always clear. A patient mentioned that his continued disclosure depended on the professional’s attitude.

So I did mention a little bit [of using natural products] to the healthcare professional and see his reaction. If he did not think so or no any response, then I won’t continue to ask. So I felt medical staff’s attitude is very important (P19).

Some conventional healthcare professionals seem to have taken advantage of the patients’ fears of the professionals’ own authority as healthcare practitioners in order to ‘discourage them from using it’, reflecting a sense of apathy and unwillingness to have open discussions about patients’ use of natural products. These patients quoted conventional healthcare professionals’ responses as follows: ‘you better watch out for it’ (P24), ‘better don’t take it’ (P35), ‘it is wasted money’ (P6), ‘you better don’t take any natural product’ (P17), and ‘the best way is to take the prescription medication’ (P28).

The second response from conventional healthcare professionals that the conventional healthcare professionals emphasised the lack of scientific evidence to support the effectiveness of natural products and hence any adverse effects was entirely their own responsibility. As patient 39 elaborated, her conventional healthcare professional commented that ‘you just take a little bit, it’s all right’ when she disclosed her use of natural products. Patient 3 reported that his conventional healthcare professional said ‘it’s all right only if you don’t feel any discomfort.’ The response of ‘you may do it’, suggests that the conventional healthcare professionals were supportive of the patient’s choice regarding the use of natural products but frequently with the condition that the natural products does not have any adverse effect or that they do not take too much of it. Therefore, if any problem were to arise, it would entirely be the patient’s own responsibility.

When my liver function levels [serum glutamic-oxaloacetic transaminase (GOT) and glutamic-pyruvic transaminase (GPT)] were down a lot… I proposed whether or not these things [natural products] had made the improvement to the healthcare professional and he didn’t think so. He indicated that these [therapies] have not been proven through clinical trials. He said ‘you may do it!’ But based on his expertise, it is not his field. (P14).

However, the patient felt that it was best to avoid further discussion of about the use of natural products because of the apparent noncommittal reply from the conventional healthcare professional that led to the conclusion that the healthcare professional did not wish to discuss the use of natural products (‘unclear communication’). From the patients’ experience and perception, there is a strong indication that tensions exist in the relationship between the patient and the conventional healthcare professional regarding this issue.

## Discussion

This study used a mixed method design to illustrate the communication of natural product usage to conventional healthcare professionals among outpatients. Our findings identified that 74% of natural product users did not disclose their usage to their healthcare professionals and this is slightly higher than the rates found in previous studies [9,26,27]. Research on the factors predicting the disclosure of using natural products to conventional healthcare professionals is limited and, consequently, there is a lack of knowledge on factors influencing the decision to disclose and not to disclose. This study identified factors previously unexplored, which have considerably refined the understanding of the reasons for the disclosure of natural product usage in conventional healthcare settings. Our findings indicated that disclosers were younger, visited health clinics roughly every 3 months, were aware of the ingredients of the natural products they were consuming and felt positive about the benefits of CAM. It is difficult to compare the findings of this study with previous studies because the factors surveyed in this study were not well documented in the literature. Among the four recent studies examining the factors associated with the disclosure of CAM use [26-29], only the factor of age was a consistent finding reflected within the current study where middle-aged people were more likely to disclose their use of natural products. It is likely that the inconsistencies in the findings in relation to the aforementioned variables are due to differences ranging from the ethnicity and culture of the samples, research design, and the range of factors looked into.

The quantitative analysis indicates variables such as knowledge of natural products and attitudes towards the benefits of CAM. People who knew what they were taking and believed in the effectiveness of natural products were curious as to whether the improvement in their health was the result of such usage. Another surprising finding is the frequency of clinical visits, in which patients who visited the clinic every three months (i.e., seasonally) were more likely to discuss their use of natural products with conventional healthcare professionals than those who visited monthly or fortnightly, mainly because those who attended clinics every three months usually have a long-term relationship with their healthcare professional, due to the chronic nature of their disease, and thus visit clinics regularly for the treatment. A possible explanation is found in other studies in which people who visited their personal healthcare professional [29] and received continuous care were more likely to disclose their use of natural products during the consultation [30,31].

The major barrier to the disclosure of using natural products to conventional healthcare professionals, as reported in the qualitative findings, was that it never crossed the patients’ minds because they felt it was safe to use these products as they were natural. However, it was also noted that many were afraid to discuss their use of the natural products as they were concerned that that would have them leave a negative impression on their conventional healthcare professionals. Additionally, patients did not feel confident in discussing the use of natural products with their healthcare professionals due to the perceived negative attitudes of the conventional healthcare professional towards the use of natural products, despite fears of adverse effects or complications resulting from combining the two forms of treatments. This is in line with the findings from the review by Robinson and McGrail [32] that patients avoid such discussions as they might garner distrust or dissatisfaction from the conventional healthcare professionals, which in turn may put their relationship at risk. One of the greatest concerns about the use of natural products is the potential for interaction with on-going conventional treatment because most patients were simultaneously engaged in conventional care and were often on numerous medications. The addition of any undisclosed nutritional supplements, herbs, or remedies may increase the risk of possible adverse reaction or interactions. Although patients reported having clarified their concerns regarding herb-drug interaction from others with some medical background, it is important to note that these people may not be qualified to give advice on specific disease management. One interesting phenomenon revealed within the in-depth interviews is that patients often detailed their conventional medication treatment to their CAM practitioners but not the other way round.

### Limitations of the study

This study has several limitations. The first limitation is that the design involved retrospective data collection, making the data subject to recall bias. A second limitation is associated with the issue of sampling bias. Quantitative data was collected to estimate the prevalence of CAM use where the non-users of CAM were subsequently excluded and this may result in potential bias of selection. Nevertheless, the ratio of patients who disclosed their use of natural products is consistent with other studies; therefore, the risk of selection bias is of little concern.

### Implementations

Having an open communication between patients and healthcare professionals with regard to the use of natural products is the key to ensuring safe implementation and integration of both medicines. Furthermore, the issues of regulation, quality, safety, and efficacy are exceedingly important to patient care. However, even though these questions are crucial in the communication process, beginning the consultation session with these questions might imply a pre-judgemental attitude towards the patients’ decision to use natural products. It is an opportunity for the conventional healthcare professionals to make a difference by approaching the interview with an open mind so as to encourage patients to disclose their use of natural products. Hence, it is necessary to establish new strategies and train the healthcare professionals in creating a supportive and collaborative atmosphere during consultations. As such, training on how to discuss the use of natural products would reduce the likelihood of patients taking potentially harmful natural products, ensure that the use of these products would be careful monitored and improve healthcare education for the patients.

In addition, serious adverse interactions could occur if the health professionals are unable to give appropriate advice because they do not have reliable knowledge of the natural products and are unfamiliar with the scientific literature on natural products. In particular, previous studies have shown that many conventional healthcare professionals feel uncomfortable discussing the effectiveness of natural products with their patients due to a lack of knowledge. In fact, the lack of sufficient knowledge and evidence-based research on CAM is a major barrier towards the conventional healthcare professionals’ ability to handle enquiries from patients about the use of natural products and appropriately refer them to reputable CAM practitioners [33,34]. There is an urgent need for studies with rigorous research methodology to establish the efficacy of the natural products. Without a thorough understanding of the pharmacodynamics of these products, it is difficult to integrate its use in congruence with conventional medicine or conduct informative education programs in the conventional medical setting. Therefore, properly designed studies to determine the effectiveness of natural products in both laboratory and clinical situations are required.

In addition, the lack of discussion with patients on the use of natural products makes it difficult for conventional healthcare professionals to recognize and report the occurrence of adverse effects and, thereby, establishing drug-herb interactions. Conversely, the synergistic effect of natural products and conventional medicines would have been missed as well, resulting in overlooked opportunities to validate such beneficial information. Therefore, the development of a standard guideline for a routine assessment of the use of natural products, a competency assessment for consultations, and an individualized plan for integrating natural products and conventional treatments that promotes safe practice is essential for all medical healthcare systems.

## Conclusions

This study provides preliminary insights into the predictors of patient’s disclosure of their use of natural products. It is an important issue as the use of natural products is an emerging trend that will have increasing impact on the delivery of healthcare with its popularity among patients. A lack of knowledge about natural products, self-administration of both medicines, and limited disclosure of such usage to conventional healthcare professionals may endanger patients. Unsupervised usage and the possibility of serious natural products-drug interaction may compromise the management of the disease and, hence, lead to an acerbation in condition as well as an increase in the utilization of the healthcare system in these patients. Since providing patients with responsible healthcare intervention from a holistic perspective is the essence of effective health care, it is important for healthcare professionals to understand the characteristics of patients who are more likely, or less likely, to disclose their use of natural products. The healthcare professionals need to take a leading role by recording the use of natural products in order to understand the effects of natural products on the patients. This would help to assess potential benefits or harm of the natural products and aid in the tracking of any side effects from its use. In addition, it is important to look into modification of the medical model of care into a more holistic model that integrates the use of natural products into the disease management programs. This will have the potential to ensure patient safety, reduce unwanted side effects, improve treatment outcomes, lower costs, and improve the professional relationship between patient-conventional healthcare professional relationships.

### Ethical approval

This project was approved by Human subject research ethics committee of Zuoying Armed Forces General Hospital.

## Abbreviations

CAM: Complementary and alternative medicine; SD: Standard deviation; OR: Odd ratios; CI: Confidence interval.

## Competing interest

The authors declare that they have no competing interests.

## Authors’ contributions

HYC and HLC were responsible for the study conception and design. HYC performed the data collection and the data analysis. HYC was responsible for the drafting of the manuscript. BS translated the qualitative data. HYC and HLC made critical revisions to the paper for important intellectual content. BS was responsible for the editing of the paper. All authors read and approved the final manuscript.

## Pre-publication history

The pre-publication history for this paper can be accessed here:

http://www.biomedcentral.com/1472-6882/13/319/prepub
